# P357: A descriptive study of maternal deaths in Algeria in 2011

**DOI:** 10.1186/2047-2994-2-S1-P357

**Published:** 2013-06-20

**Authors:** N Keddad

**Affiliations:** 1 Ministry of Health, Population and Hospital Reform, Algiers, Côte d'Ivoire

## Introduction

In our country, despite a substantial increase in the assisted delivery rate to 97%, the maternal mortality rate remains high. 70% of maternal deaths occur during delivery and the postpartum period.

## Objectives

Perform a clinical audit of maternal deaths to identify causes to reduce the risk of maternal death, for care based on quality assurance.

## Methods

This audit was performed through the use of a standard questionnaire on all deaths occurring in 2011; the questionnaire was sent to health administrators of the 48 health departments of Wilayas. Data analysis was performed on the software SSPRO.

## Results

Out of 220 maternal deaths, 93% occurred in hospital, 26% following an evacuation in an emergency. Some causes include hemorrhage 31.4%; HTA / complication 12.7%; cardiovascular 12.3%; infections 6.4%. The ages ranged from 19 to 55 years with an average of 32.9 years. The Deaths were most frequent at first pregnancy (39%) and at fourth or later pregnancy (32%). Outcomes for fetuses were live birth in 44.5%, stillbirth in 33.6%, and abortion in 2.7%.

## Conclusion

Access to essential obstetric care quality was limited: no hierarchy of levels of care, lack of organizational standards and functional at the hospital; evacuations late coupled with inefficient management of obstetric emergencies. Weight of the high risk of cardiovascular disease.

## Disclosure of interest

None declared

